# An efficient constructive heuristic for the rectangular packing problem with rotations

**DOI:** 10.1371/journal.pone.0295206

**Published:** 2023-12-28

**Authors:** Xusheng Zhao, Yunqing Rao, Peng Qi, Qianhang Lyu, Piaoruo Yang, Shoubo Yu

**Affiliations:** 1 School of Mechanical Science & Engineering, Huazhong University of Science and Technology, Wuhan, Hubei, China; 2 State Key Laboratory of Intelligent Manufacturing Equipment and Technology, Huazhong University of Science and Technology, Wuhan, Hubei, China; National Institute of Technology Silchar, India, INDIA

## Abstract

The rectangular packing problem has been extensively studied over the years due to its wide application in industry. However, most of the research efforts are devoted to positioning techniques of the rectangles for various problem variants, the efficient implementation of the packing procedure is relatively less studied. In this paper, we propose an efficient constructive algorithm for the rectangular packing problem with rotations. We design a preprocess procedure with four data structures to store the information used for item selection. The gaps on the skyline are categorized into three types according to their associated edges for the placement procedure, during which the item is searched and packed in a descending order of the fitness value. The entire constructive phase takes a time complexity of O(*n*log*n*). For the packing improvement phase, we optimize the packing through random perturbation on the sequence and orientation of the item. Three classes of stochastic problems are generated ranging from small-scale to extra-large-scale, the recorded running time confirms the efficiency of the proposed algorithm. We also test the proposed algorithm on the benchmark problem C21, N13, NT, Babu and CX, the computational results show that it delivers a good performance.

## 1. Introduction

Given a group of large objects with regular shape and homogeneous material, designing an assortment of the small items accommodated by the large objects to minimize the waste is the classic cutting and packing problem [1]. Additional constraints in industrial applications may add properties to the cutting and packing problem, which defines a wide range of problem variants. For example, the small items can be rectangles, circles, polygons or even contour shapes with curved edges, and the large objects may have concave areas or defective parts that cannot be used for placement [2].

Usually, the computational time allowed for any specific problem depends on the material utilization and the task urgency, thus, most of the solutions attempt to make a trade-off between solution quality and the time consumption [3]. From a mathematical point of view, cutting and packing are closely related as they indicate the same computing process, they also share the same target that the predefined objective should be achieved with the fewest possible resources or the limited resources should be used to generate the best possible results [4].

The rectangular packing problem is categorized as a sub-type of the cutting and packing problem, where the items are rectangles and should be placed exactly into the container without overlap [5]. There are two most documented variants of the rectangular packing problem, the multiple bin packing problem and the strip packing problem. The former arranges a group of identical-sized or variable-sized bins and the items are supposed to fill the bins as much as possible [6–8], the later defines a container holding an open dimension which needs to be minimized through elaborate placement of all the given items [9–11].

Since real application may focus on more complicated scenarios in the field of production planning, scheduling, routing and loading, multi-objective optimization in combination with packing has attracted an increasing amount of attention as well. Wu studied a variant in which the ratio of the two dimensions of the rectangular layout should be kept within given range and predefined central rectangle should be placed near the center of the layout [12]. Fernández analyzed the approach to extend memetic algorithms to solve a the two-dimensional bin packing problem with requirements on loading balance [13]. Queiroz dealt with the two dimensional strip packing problem where the gravity center of the item must lie in a safety region and the given maximum tolerable weight which the item could bear must be satisfied [14]. Gajda studied a container loading problem handling a group of constraints simultaneously, including loading priorities, load bearing and balancing, unloading order, weight limits, cargo stability and positioning constraints [15].

A few exact methods have been proposed to solve the rectangular packing problem in recent years, the algorithms are mainly based on integer programming [16, 17] and dynamic programming [18]. While the exact methods are often used to solve problems of small-scale, most of the literature adopts the heuristic approaches. Burke [3] proposed a best fit (BF) strategy, the algorithm examines the lowest available space and always places the rectangle which matches the space. BF is reported to have made significant progress in terms of packing quality as well as computational efficiency, it is further studied over the years in the placement techniques [19, 20] or the improvement methods [21, 22].

Another recent attempt to solve the packing problem is to automate the design of heuristics, since the problem specific methods may have insufficient generality for other problems within a domain [23]. López-Camacho et al. developed a selection hyper-heuristic approach which constructs the solution incrementally, and each step contains a forward check to ensure the solution quality. Their model automatically selects the best heuristic for a given instance and the results are comparable to single heuristic [24].

As BF executes with the dynamic selection of the item during each placement, trivial implementation naturally takes a time complexity of O(*n*^2^). Although many heuristics with the BF architecture have been adopted to solve the packing problems, there are considerably few studies researching the efficient implementation of the packing procedure. The first research considering the efficient implementation of the BF heuristic is reported by Imahori & Yagiura, they stored the skyline with a heap and a doubly linked list, then, they used the balanced binary search tree to search for the fittest rectangle [25]. Wei et al. proposed a scoring rule based BF heuristic for the fixed orientation variant of the strip packing problem, where the scoring rule evaluates the matching edges between the item and the skyline, and they used a segment tree to search for the item in particular placement [26].

Inspired by Wei’s study [26], this paper presents an efficient constructive heuristic for the rectangular strip packing problem, in which the rectangles are allowed with the rotation of 90°. The constructive phase of the packing is initialized with a preprocess in which four data structures are designed to store the necessary information required by item selection. Then, the placement of item is implemented piece by piece, and the four data structures are updated continuously with the item selection. An optimization algorithm with random perturbation is provided for the packing improvement. The main contributions of this paper are: (i) an efficient implementation method of the rectangular constructive phase; (ii) an optimization algorithm to improve the packing quality; (iii) comparison between the efficient implementation and the original implementation on a group of packing problems from small-scale to extra-large-scale; and (iv) performance evaluation on a set of benchmark problems, which demonstrates that the proposed method provides satisfactory packing results.

The rest of the paper is organized as follows. In section 2, we provide the preliminaries of the study, including the best fit heuristic architecture and the segment tree used for item searching. In section 3, we propose the efficient rectangular packing heuristic for the constructive phase and design an optimization algorithm for the improvement phase. We present the computational experiments in section 4 and summarize the conclusions in section 5.

## 2. Rectangular selection strategy

The problem solved in this paper is defined in the same way as [27], i.e., a container with a fixed width denoted by *W* and an open dimension is provided to accommodate the rectangles. Let the number of the rectangles to be packed be *n*, we denote the length of each rectangle by *l*_*i*_, width by *w*_*i*_, *i*∈[1, *n*]. The packing length is denoted by *L’*. The rectangles are allowed of the rotation of 90°.

We follow the architecture of the BF heuristic [3] and use the fitness evaluation rule [26] for the item selection. We always choose the bottom left skyline as the placement position. Then, the associated gap on the boundary [27] determines the shape of the next item to be placed. The fitness evaluation rule is used to select the most suitable rectangle. The fitness value is computed as the number of identical dimensions between the item and the gap, if there is an edge of the item which aligns with that of the gap, the fitness value is increased by 1. Then, the fitness value of the placement could 3, 2, 1 or 0.

If there is a tie, the earliest item in the sequence which has not been placed will be chosen. Then, under specific settings, the item selection is transferred to the range minimum query problem, where the item holding the minimal index is supposed to be picked out. The Segment tree [28, 29] is used to solve the range minimum query problem. Suppose the given range contains *n’* elements. The segment tree is constructed through the decomposition of the large range into small units. Then, the node is iteratively assigned with the minimum value of the split range, from the leaf to the parent. The construction of the segment tree takes a time complexity of O(*n’*log*n’*), and both the query of a particular range and the update of single element take O(log*n’*).

## 3. Efficient rectangular packing heuristic

In this section, we present the efficient heuristic for the rectangular strip packing problem. We propose the efficient implementation of the constructive phase in section 3.1 and design a optimization algorithm for the improvement phase in section 3.2.

### 3.1. Packing constructive algorithm

The constructive phase is composed of the preprocess procedure and the placement procedure. The preprocess procedure defines the necessary data structures used throughout the packing, and the placement procedure implements the placement of the *n* items piece by piece.

**Fig 1:**
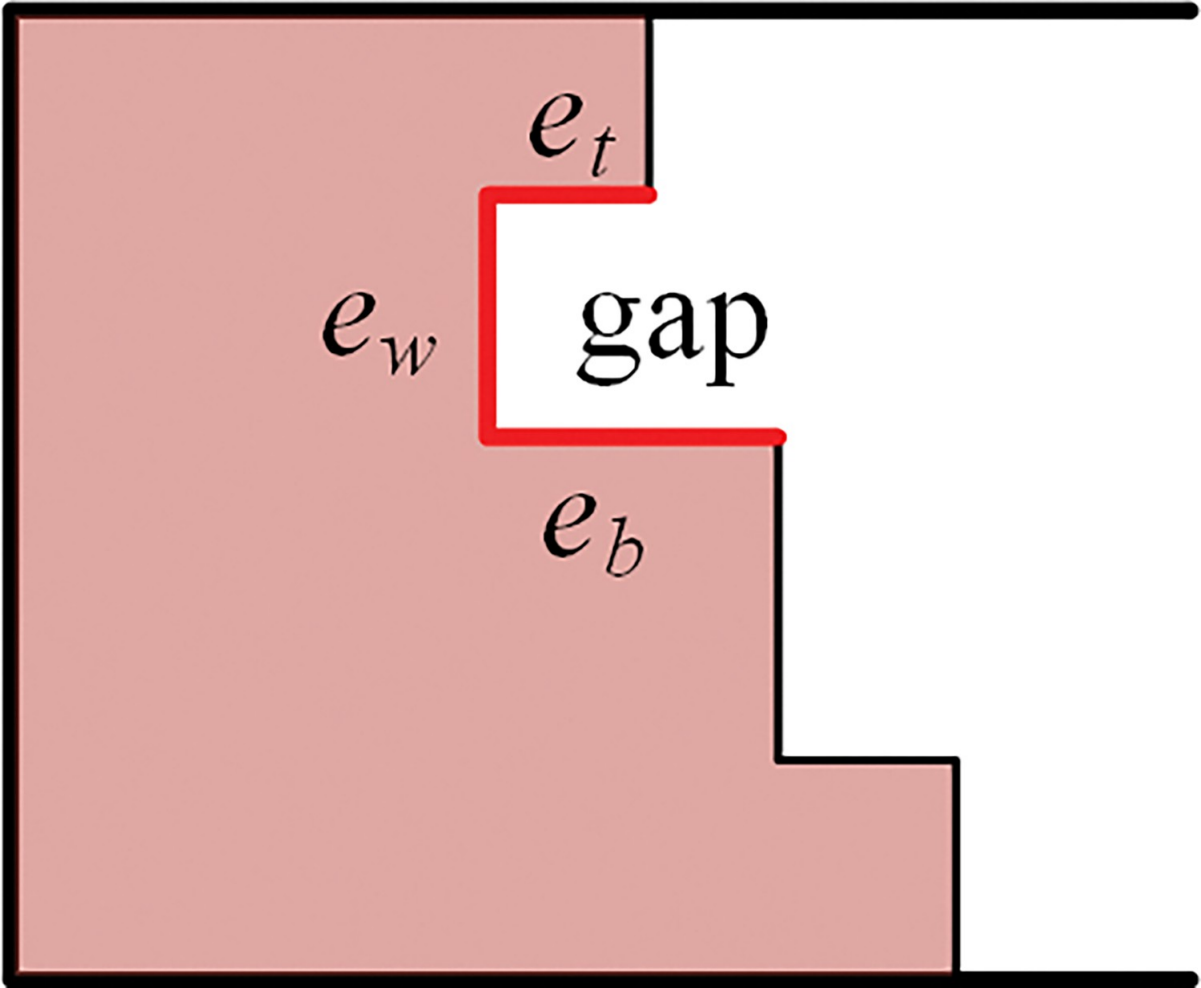
Illustration of the gap on the boundary.

We denote the top edge of the bottom left gap by *e*_*t*_, the side edge which represents the width of the gap by *e*_*w*_, and the bottom edge by *e*_*b*_. as shown in Fig 1, the gap is marked with highlight. In this section, the fitness value of the placement is defined as the number of alignments between the item and the three edges of the gap.

#### 3.1.1 Preprocess

We design four data structures to search for the particular items, which are denoted by *D*_*1*_, *D*_*2*_, *D*_*3*_ and *D*_*4*_, respectively. We list the type of basic data structure contained in *D*_*1*_–*D*_*4*_ and the target placement of each data structure in Table 1.

**Table 1:** Data structure for item search.

	Contained date structure type	Search target
*D*_*1*_	hash table, list	fitness = 3, fitness = 2
*D*_*2*_	hash table, list	fitness = 1 (with *e*_*w*_ alignment)
*D*_*3*_	hash table, list, segment tree	fitness = 1 (with *e*_*b*_ alignment)
*D*_*4*_	list, segment tree	fitness = 0

In the data structure *D*_*1*_, we use a hash table and a group of lists to store the items. The key represents the size of each type of rectangle, it is recorded by (*l*_*i*_, *w*_*i*_), i.e., a tuple of the length and width of the rectangle, and the value is represented by a list to store the given sized item. *D*_*1*_ is built by traversing the items. When a new size type of the item is met, a new key will be added to the hash table, and a list is initialized with the item specified as the first element. If an item with the size already stored in the hash table, the relevant list will be found through the key, then, the item will be appended to the list.

An example is provided for the construction of the four data structures. Suppose there are nine items to be packed, we denote them by *r*_*1*_–*r*_*9*_ and their sizes are (2, 3), (4, 5), (3, 2), (6, 8), (4, 5), (6, 10), (6, 3), (6, 5) and (6, 7). The first item with size (2, 3) will add a key of (2, 3) to *D*_*1*_, and it becomes the first element in the relevant list, then, *D*_*1*_ = {key: (2, 3), value: [*r*_*1*_]}. The second item will update *D*_*1*_ in the same way, so will the third item and the fourth item. After the update by the fourth item, *D*_*1*_ = {key: (2, 3), value: [*r*_*1*_]; key: (4, 5), value: [*r*_*2*_]; key: (3, 2), value: [*r*_*3*_]; key: (6, 8), value: [*r*_*4*_]}. When it comes to the fifth item, there already exists the key (4, 5), so no key will be added to *D*_*1*_, and the fifth item will be appended to the list with key (4, 5), then, *D*_*1*_ = {key: (2, 3), value: [*r*_*1*_]; key: (4, 5), value: [*r*_*2*_, *r*_*5*_]; key: (3, 2), value: [*r*_*3*_]; key: (6, 8), value: [*r*_*4*_]}. When the traversal of the nine items is over, *D*_*1*_ = {key: (2, 3), value: [*r*_*1*_]; key: (4, 5), value: [*r*_*2*_, *r*_*5*_]; key: (3, 2), value: [*r*_*3*_]; key: (6, 8), value: [*r*_*4*_]; key: (6, 10), value: [*r*_*6*_]; key: (6, 3), value: [*r*_*7*_]; key: (6, 5), value: [*r*_*8*_]; key: (6, 7), value: [*r*_*9*_]}.

In the data structure *D*_*2*_, another hash table is used to store the items. Unlike *D*_*1*_, the key represents the type of the single dimension of the rectangle, which is recorded with either *l*_*i*_ or *w*_*i*_, and the value is represented by a list to store the item holding the given dimension. We continue with the example of the nine items presented above. The first item has the dimension of 2 and 3, then, a key of 2 and another key of 3 will be added to *D*_*2*_, and the first item becomes the first element both in the relevant list of key 2 and the relevant list of key 3, i.e., *D*_*2*_ = {key: 2, value: [*r*_*1*_]; key: 3, value: [*r*_*1*_]}. The rest of the seven items will update *D*_*2*_ in the same way. When the traversal of the nine items is over, *D*_*2*_ = {key: 2, value: [*r*_*1*_, *r*_*3*_]; key: 3, value: [*r*_*1*_, *r*_*3*_, *r*_*7*_]; key: 4, value: [*r*_*2*_, *r*_*5*_]; key: 5, value: [*r*_*2*_, *r*_*5*_, *r*_*8*_]; key: 6, value: [*r*_*4*_, *r*_*6*_, *r*_*7*_, *r*_*8*_, *r*_*9*_]; key: 8, value: [*r*_*4*_]; key: 10, value: [*r*_*6*_]; key: 7, value: [*r*_*9*_]}.

**Fig 2:**
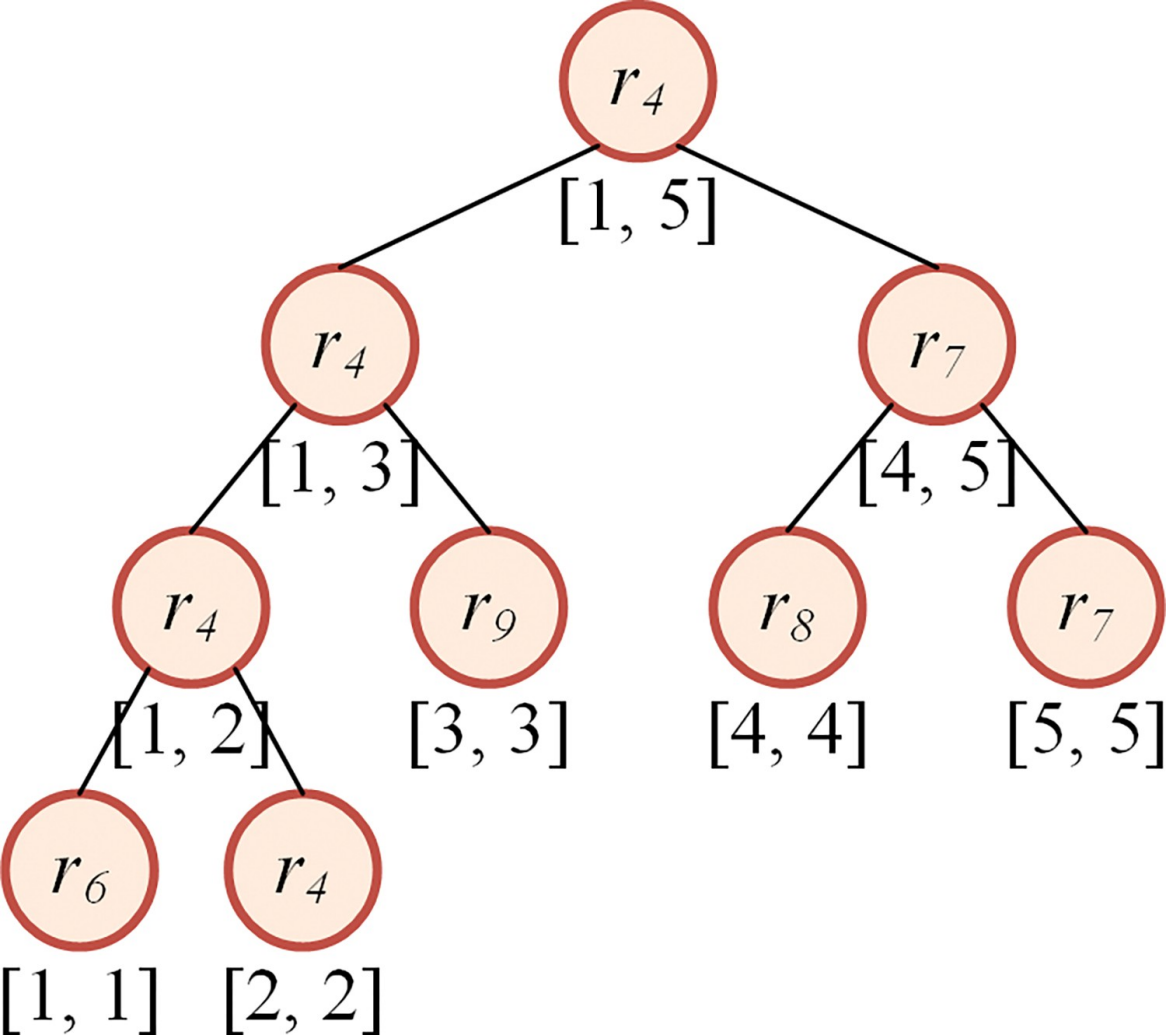
An example of segment tree with node indicating the minimum item index.

The data structure *D*_*3*_ is generated from *D*_*2*_. We make a copy of *D*_*2*_, then, we sort the items in the list of each key by the other dimension of the item in the descending order. Then, a segment tree is built according to the sorted sequence. The hash table, sorted sequence and the segment tree comprise *D*_*3*_. The node of the segment tree represents the item with the minimum index in the given range. For example, the list of key 6 has five items namely *r*_*4*_, *r*_*6*_, *r*_*7*_, *r*_*8*_ and *r*_*9*_, and the other dimensions of them are 8, 10, 3, 5, 7, respectively. Then, the list would be [*r*_*6*_, *r*_*4*_, *r*_*9*_, *r*_*8*_, *r*_*7*_] after sorting. The segment tree is built as shown in Fig 2.

For the data structure *D*_*4*_, we sort the items by the minimal size of their two dimensions in the descending order. Another segment tree is built based on the sorted sequence, with the node still representing the item with the minimum index in the given range. For example, the minimal sizes of the nine items *r*_*1*_–*r*_*9*_ are 2, 4, 2, 6, 4, 6, 3, 5, 6, respectively, then, the list after sorting would be [*r*_*4*_, *r*_*6*_, *r*_*9*_, *r*_*8*_, *r*_*2*_, *r*_*5*_, *r*_*7*_, *r*_*1*_, *r*_*3*_]. The segment tree is built in the same way as that in *D*_*3*_, with the associated range containing all the 9 items.

We sort the items with merge sort in *D*_*3*_ and *D*_*4*_, during which we record the position of each item in the sorted sequence.

Since the skyline is continually updated with each placement, if a skyline does not have enough space for further placement, the skyline will be removed and merged into the neighbor skylines. The space for further placement is judged by the size of the skyline and the minimum dimension of the rest items. Hence, we have to continually track the smallest sized item in order to check if it has been placed or not. We denote the minimum dimension of the rest items by *lw*_*min*_. We use another hash table denoted by *H*_*lwmin*_ to store all the items, and we make a copy of the sorted sequence in *D*_*4*_. Then, we design a pointer denoted by *P*_*lwmin*_ to locate the last item in the sequence, and *lw*_*min*_ is initialized with the minor value of the two dimensions of the item pointed by *P*_*lwmin*_.

As presented in Table 1, among the four data structures *D*_*1*_–*D*_*4*_, *D*_*1*_ is used to search for the full matching placement, i.e., the item which could produce a fitness value of 3 or 2. If none of the remained items satisfy the placement with the fitness value of 3 or 2, *D*_*2*_ and *D*_*3*_ will be used to search for the item which has one dimension alignment with the gap. If none the remained items could not create the placement with non-zero fitness value, *D*_*4*_ will be used to search for the item which could be accommodated by the gap. The details of the item selection will be described in section 3.2.

The data structure *D*_*1*_ is built with the traversal of the items, both the addition of new key and the search for the existing key take the constant time, so does the element addition in the relevant list. Then, the construction of *D*_*1*_ takes O(*n*). The construction of *D*_*2*_ is similar to *D*_*1*_ and has the time complexity O(*n*). The merge sort takes O(*n*log*n*), and the construction of the segment tree takes O(*n*log*n*), then, the constructions of *D*_*3*_ and *D*_*4*_ take O(*n*log*n*). Hence, the time complexity of the preprocess is O(*n*log*n*).

#### 3.1.2. Efficient implementation of rectangular constructive algorithm

**Fig 3:**
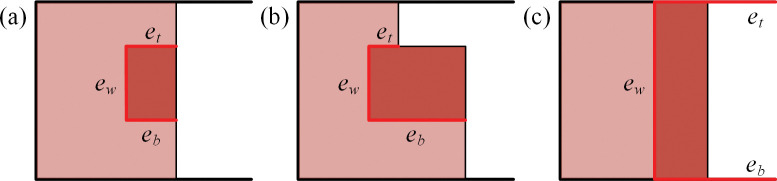
Placement between the item and the gap. (a) Type 1: *e*_*t*_ = *e*_*b*_. (b) Type 2: *e*_*t*_ ≠ *e*_*b*_. (c) Type 3: *e*_*t*_ = *e*_*b*_ = ∞.

According to the position of the gap, we divide the placements of the rectangles into three types, as illustrated in Fig 3.

In the first type, the gap is positioned inside the container, and its top edge and bottom edge have the same size, i.e., *e*_*t*_ = *e*_*b*_. The placement could have a maximum fitness value of 3 if the item has the full matching with the gap, as shown in Fig 3(A). Otherwise, the fitness value would be 1 if the item only aligns with one edge of the gap. If the item has a completely different shape compared with the gap that none of its edges could align with the gap, then, the fitness value is 0.

In the second type, the gap is also positioned inside the container, with its top edge and bottom edge shaped with different size, i.e., *e*_*t*_ ≠ *e*_*b*_. The maximum fitness value of the placement is 2 if the item could align with *e*_*w*_ and *e*_*t*_ or *e*_*w*_ and *e*_*b*_. as shown in Fig 3(B). The fitness value of 1 and 0 is produced by the placement similar to the first case.

The third type is relatively special which represents the case that none of the items have been packed, or the case that the packed items exactly share the same boundary. In this type, the skyline fills the fixed dimension of the container, and both *e*_*t*_ and *e*_*b*_ could extend without limitation. The fitness value could be 1 only if one dimension of the item is equal to the width of the container.

**Fig 4:**
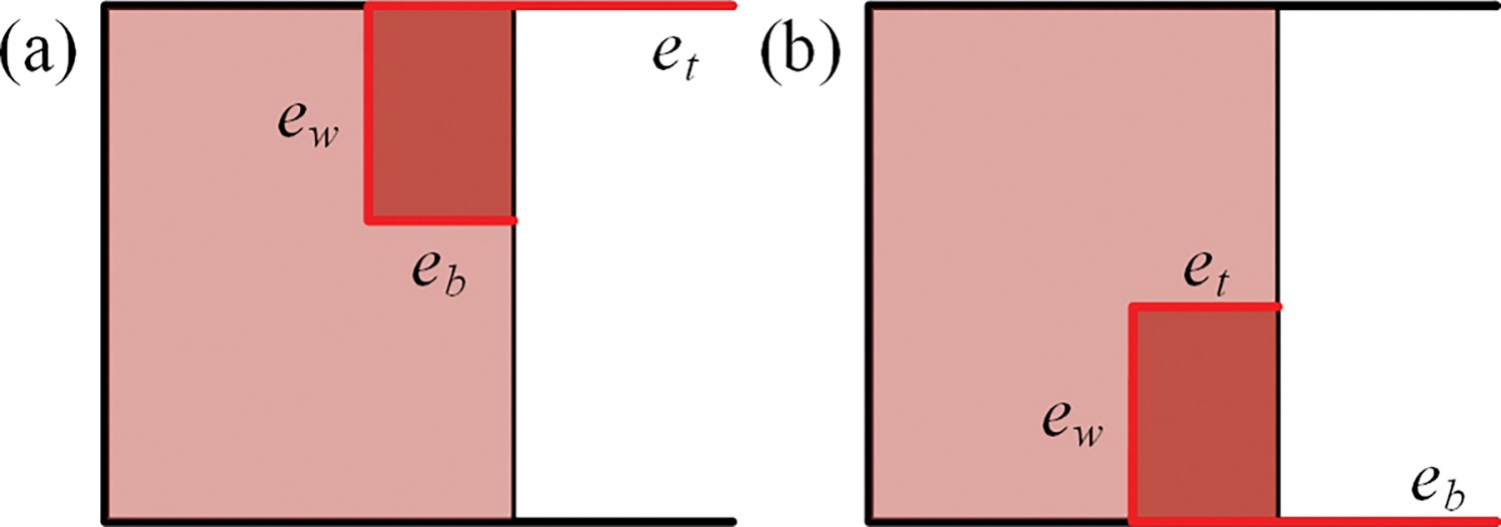
Placement at the top or the bottom of the container. (a) Gap at the top, *e*_*t*_ = ∞. (b) Gap at the bottom, *e*_*b*_ = ∞.

Note that the case in which the gap is positioned on the top of the container (as shown in Fig 4(A)) or the case in which the gap is positioned on the bottom of container (as shown in Fig 4(B)) could be categorized into the first type.

Whether an item could be placed at the gap is decided by the width of the gap. If one of the item dimensions is smaller than *e*_*w*_, then, the item could be placed at the gap, either with a rotation of 0° or 90°.

We initialize the packing length *L’* with 0. As there might be more than one item that could produce the placement with non-zero fitness value, we use three lists to store the item in the placement with the fitness value of 3, 2, 1, and they are denoted by *L*_*f = 3*_, *L*_*f = 2*_, *L*_*f = 1*_, respectively. The efficient constructive algorithm is presented in algorithm.

Each placement starts from the gap shaped by the bottom left skyline (line 3, 4), and the placements under the gap type 1 (line 5–15), the gap type 2 (line 16–26) and the gap type 3 (line 27–33) are addressed individually.

If the gap belongs to the type 1, the item with fitness value 3 will be searched first. The item should be shaped with both the dimension of *e*_*b*_ and *e*_*w*_. We search for the key of (*e*_*b*_, *e*_*w*_) in *D*_*1*_, if the key exists, enter the relevant list of item, and we select the first item without mark (i.e., it has not been packed) and store the item in *L*_*f = 3*_. We also search for the key of (*e*_*w*_, *e*_*b*_) in *D*_*1*_, which represents the placement of the item with size (*e*_*w*_, *e*_*b*_) under a rotation of 90°, and we store the item in *L*_*f = 3*_ (line 7). If *L*_*f = 3*_ ≠ Ø, we choose the item with the minor index in *L*_*f = 3*_ as the one to be packed (line 9). Otherwise, there does not exist the placement of fitness value 3, we continue with the search for the placement of fitness value 1.

**Algorithm 1**. Packing constructive algorithm

1. Initialize *L’* and the skyline

2. **for** *i* = 1 to *n* **do**:

3. Choose the bottom left skyline

4. Compute *e*_*t*_, *e*_*w*_, *e*_*b*_ of the gap

5. **if** the gap belongs to type 1:

6.   Reset the list *L*_*f = 3*_, *L*_*f = 1*_

7.   Search for item with fitness value 3 and store the result in *L*_*f = 3*_

8.   **if** *L*_*f = 3*_ ≠ Ø:

9.  choose the item with minor index in *L*_*f = 3*_.

10.   **else**:

11.  Search for item with fitness value 1 and store the result in *L*_*f = 1*_

12.  **if** *L*_*f = 1*_ ≠ Ø:

13.    choose the item with minor index in *L*_*f = 1*_.

14.  **else**:

15.    Search for item with fitness value 0 and choose the item

16. **if** the gap belongs to type 2:

17.   Reset the list *L*_*f = 2*_, *L*_*f = 1*_

18.   Search for item with fitness value 2 and store the result in *L*_*f = 2*_

19.   **if** *L*_*f = 2*_ ≠ Ø:

20.  choose the item with minor index in *L*_*f = 2*_.

21.   **else**:

22.  Search for item with fitness value 1 and store the result in *L*_*f = 1*_

23.  **if** *L*_*f = 1*_ ≠ Ø:

24.    choose the item with minor index in *L*_*f = 1*_.

25.  **else**:

26.    Search for item with fitness value 0 and choose the item

27. **if** the gap belongs to type 3:

28.   Reset the list *L*_*f = 1*_

29.   Search for item with fitness value 1 and store the result in *L*_*f = 1*_

30.   **if** *L*_*f = 1*_ ≠ Ø:

31.  choose the item with minor index in *L*_*f = 1*_.

32.   **else**:

33.  Search for item with fitness value 0 and choose the item

34. Update data structure *D*_*1*_–*D*_*4*_

35. Compute the packing length *L’*

36. Relocate the pointer *P*_*lwmin*_ and reset the minimum dimension *lw*_*min*_

37. Update the skyline

38. **return** *L’*

For the placement of fitness value 1, we search for the key *e*_*w*_ in *D*_*2*_ and store the item in *L*_*f = 1*_, which represents the alignment between *e*_*w*_ of the gap and the item. We also search for the key *e*_*b*_ in *D*_*3*_, which represents the alignment between *e*_*b*_ of the gap and the item. In the relevant list of key *e*_*b*_, the item has to satisfy an additional requirement that the dimension other than *e*_*b*_ should be smaller than *e*_*w*_. We use the binary search to find the position of the first item which has the dimension smaller than *e*_*w*_, we denote the position by *p*_*D3*_, then, the items in the range from *p*_*D3*_ to the end of the list could meet the dimension requirement. The segment tree in *D*_*3*_ (presented in section 3.1.1) is used to find the item with the minimum index in the given range, and the item will also be stored in *L*_*f = 1*_ (line 11). To continue with the example presented in section 3.1.1, we suppose that *e*_*b*_ = 6 and *e*_*w*_ = 9. Then, the search for the key *e*_*b*_ in *D*_*3*_ results in the list [*r*_*6*_, *r*_*4*_, *r*_*9*_, *r*_*8*_, *r*_*7*_], and the additional requirement on the other dimension limits the available item within the range [*r*_*4*_, *r*_*9*_, *r*_*8*_, *r*_*7*_]. In this case, the item *r*_*4*_ is selected since it has the minimum index, and it is stored in *L*_*f = 1*_. If *L*_*f = 1*_ ≠ Ø, we choose the item with the minor index in *L*_*f = 1*_ as the one to be packed (line 13). Otherwise, there does not exist the placement of fitness value 1, we search for the placement of fitness value 0 (i.e., the item with the minimal index which could be placed at the gap).

For the placement of fitness value 0, we use the binary search to find the position of the first item which has the dimension smaller than *e*_*w*_ in the list of *D*_*4*_, we denote the position by *p*_*D4*_, then, the items in the range from *p*_*D4*_ to the end of the list could be placed at the gap. The segment tree in *D*_*4*_ is used to find the item with the minimal index in the given range (line 15).

If the gap belongs to the type 2 or the type 3, the operation is similar to that of type 1, with priority given to the placement of the larger fitness value.

After the item selection, the placement orientation of the item is determined by the match between its dimension and the shape of the gap. Besides, each placement of the chosen item will update the four data structures.

For the update of *D*_*1*_, group the length *l*_*i*_ and the width *w*_*i*_ of the selected item as the key (*l*_*i*_, *w*_*i*_) and search the key in *D*_*1*_, then the relevant list could be returned. As each item is appended to the end of the list in the preprocess, the items are naturally sorted in an increasing order of the index. We use binary search to search for the index of the item and we get the position, then, we mark this position as the item has been placed.

The update of *D*_*2*_ is similar to *D*_*1*_, we use both the length *l*_*i*_ and the width *w*_*i*_ of the selected item as the key and get the relevant list individually. Then, we use the binary search to find the item and mark the item as used.

**Fig 5:**
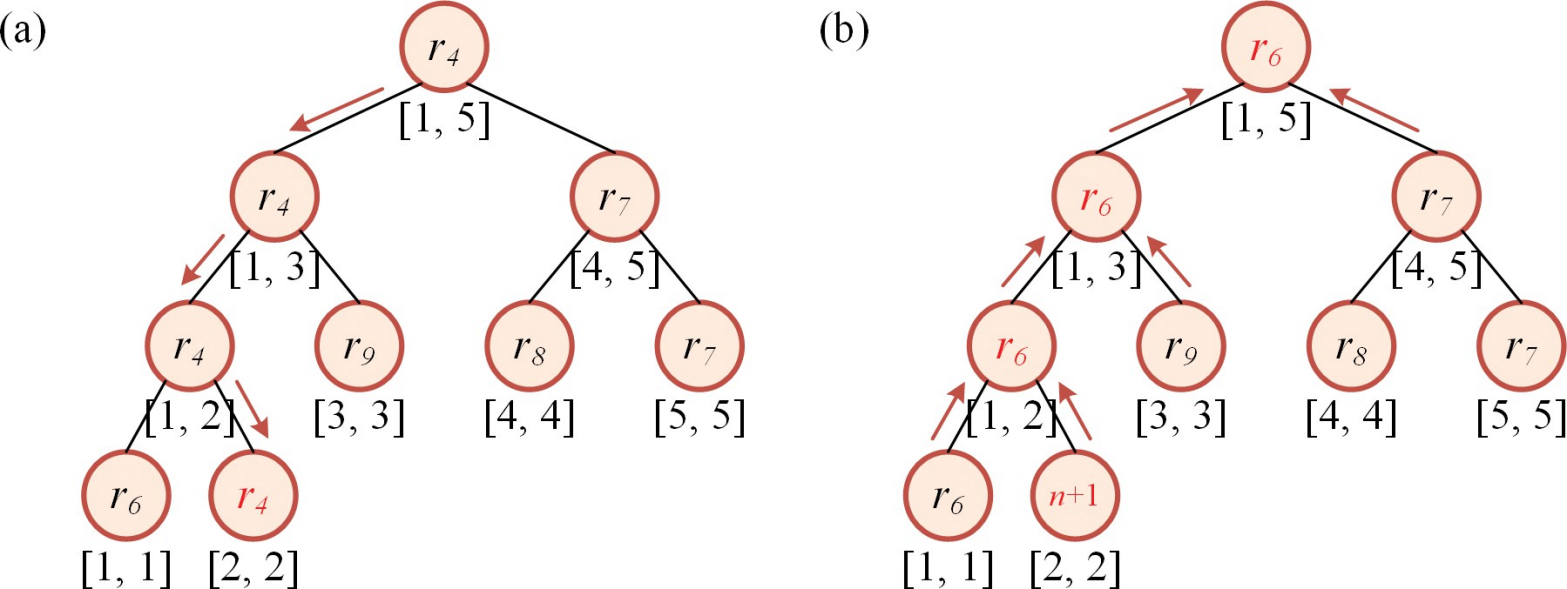
Update of the segment tree. (a) Leaf node track. (b) Node value update.

For the update of *D*_*3*_, as the hash table in *D*_*3*_ has the same key as that in *D*_*2*_, we already get the position of the chosen item in the relevant list in *D*_*2*_, and we have recorded the position of each element of the list in the sorted sequence (mentioned in section 3.1.1), then, the position of the chosen item in the relevant list in *D*_*3*_ could be computed. The leaf node will be tracked recursively along the direction from the root to the associated child, and its value is replaced with *n*+1, then, the item will not be selected in later steps in the range query on the tree. For example, when the item *r*_*4*_ is selected, it can be computed that *r*_*4*_ lies in the second position of the relevant list, with which the leaf node in the segment tree is tracked and found, as shown in Fig 5(A). Then, all the nodes along the track are updated, the minimal value of each node and its sibling node will replace that of the parent node, as shown in Fig 5(B).

The update of *D*_*4*_ is similar to *D*_*3*_, we replace the associated node in the segment tree with value of *n*+1.

We also update the minimum dimension of the rest items. Each selected item will be removed from the hash table *H*_*lwmin*_. Then, if the item being located by the pointer *P*_*lwmin*_ is still in *H*_*lwmin*_, it means the item with the minimum dimension has not been placed, thus, the minimum size *lw*_*min*_ will remain unchanged. If the item being located by *P*_*lwmin*_ is not in *H*_*lwmin*_, *P*_*lwmin*_ will move towards the beginning of the sequence one by one, until it reaches the item that is still in *H*_*lwmin*_. Then, *lw*_*min*_ will be replaced by the minimum dimension of the pointed item.

The placement procedure is composed of three major steps, namely the selection of the fittest item, the update of the data structures, and the update of the minimum size *lw*_*min*_ of the item. The search through hash table takes constant time, while the query on the segment tree takes O(log*n*), then, the selection of the fittest item takes O(log*n*). The update of the four data structures also takes O(log*n*) due to the update of the node in the segment tree. Thus, these two steps in the traversal take O(*n*log*n*). For the update of *lw*_*min*_, as the pointer *P*_*lwmin*_ purely moves towards the beginning of the sorted sequence throughout the packing of the *n* items, it takes O(*n*). Hence, the placement procedure takes O(*n*log*n*), and the time complexity of the entire constructive phase is O(*n*log*n*).

**Fig 6:**
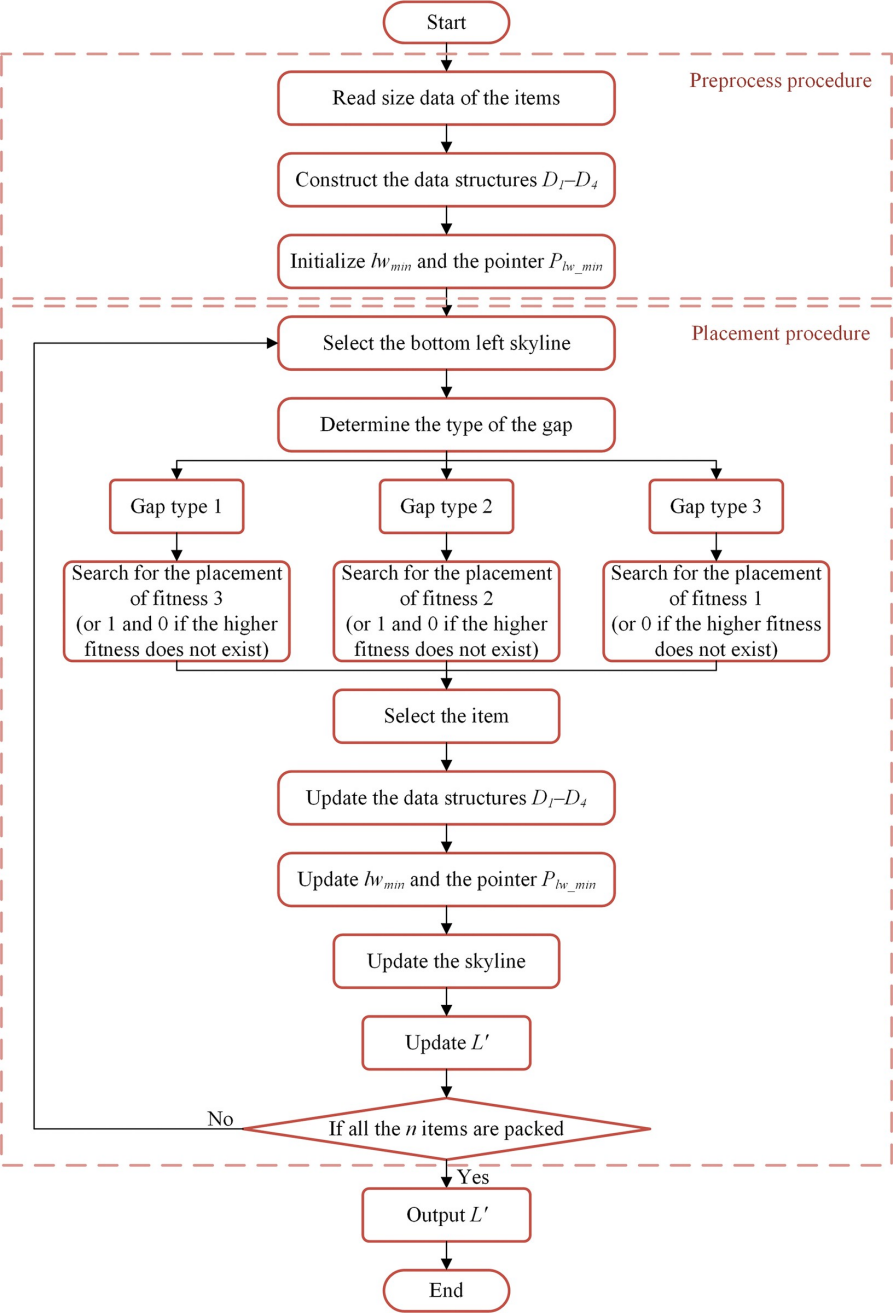
Flowchart of the constructive phase.

The procedure of the constructive phase is illustrated in Fig 6, the preprocess procedure (presented in section 3.1.1) and the placement procedure (presented in this section) are marked separately. The four data structures *D*_*1*_–*D*_*4*_ and the pointer *P*_*lwmin*_ are initialized in the preprocess procedure. Then, each placement starts from the bottom left skyline and the type of the gap is decided. The item is selected according to the fitness value from largest to smallest, and *D*_*1*_–*D*_*4*_ as well as *P*_*lwmin*_ are updated.

### 3.2. Packing optimization algorithm

We design the optimization algorithm for the improvement phase. Since the rectangles are allowed with the rotation of 90°, the solution is composed of the sequence and the orientation of the *n* items. We adopt the idea proposed by Wei et al. [26] and adapt it to the optimization on both the sequence and the orientation.

We denote the sequence and orientation of the *n* items by *S* and *O* respectively. The sequence, orientation and the packing length of the best solution are denoted by *S*_*opt*_, *O*_*opt*_ and *L’*_*opt*_ respectively.

A group of solutions is used to explore the neighborhood of the optimal solution. We denote the number of the solutions by *N*_*g*_, the sequence of the *i*-th solution by *S*_*i*_ and the orientation the *i*-th solution by *O*_*i*_, *i*∈[1, *N*_*g*_]. The packing optimization algorithm is presented in algorithm 2.

**Algorithm 2**. Packing optimization algorithm

1. Read the size data of the item

2. **for** *i* = 1 to *N*_*group*_ **do**:

3. Initialize *S*_*i*_ and *O*_*i*_

4. Compute the packing length *L’*_*i*_ (with algorithm 1)

5. Select *S*_*i*_, *O*_*i*_ and *L’*_*i*_ of the best solution as *S*_*opt*_, *O*_*opt*_ and *L’*_*opt*_

6. **for** *iter* = 1 to *t* **do**:

7. **for** *i* = 1 to *N*_*group*_ **do**:

8.  Regenerate *S*_*i*_ and *O*_*i*_ through *S*_*opt*_ and *O*_*opt*_

9.  Compute the packing length *L’*_*i*_ (with algorithm 1)

10. Update *S*_*opt*_, *O*_*opt*_ and *L’*_*opt*_

11. **return** *L’*_*opt*_

Algorithm 1 is invoked to pack the items under the given sequence and the given orientation, with the packing length *L’* of the *i*-th solution returned (line 4, 9). The group of solutions are initialized in line 2–4, the sequence *S*_*i*_ is initialized in the descending order of the perimeter of the item, and the orientation *O*_*i*_ is initialized with random rotation of 0° or 90° (line 3). Then, the solution with the minimal packing length is selected as the optimal solution (line 5).

The group of solutions is updated continually in the iterations (line 6–10). Each solution is updated through an operator of random swap implemented on the optimal sequence *S*_*opt*_ or an operator of random orientation change on the optimal orientation *O*_*opt*_ (line 8). The optimal solution will be replaced with new solution which produces the smaller packing length (line 10).

## 4. Experiments and discussion

In this section, we generate a group of stochastic problems to testify the running time of the proposed algorithm. Then, we test the proposed algorithm on a set of benchmark problems. The algorithm is coded in python and tested under the computational environment of 2.3GHz CPU and 16 GB RAM without multithreading.

We design the stochastic problems in which the number of item is set with 10^*t*^, where *t*∈[1,6], representing the packing problems from small-scale to extra-large-scale. We denote these six stochastic problems by P1–P6, respectively. Three classes of value ranges are set on the item dimension, namely [10, 100], [10, 500] and [10, 1000], and we denote them by class1, class2 and class3 respectively. Both the dimensions of each item are randomly selected integers within the given range.

We list the time consumption of both the original implementation (time complexity O(*n*^2^)) and the proposed efficient implementation (time complexity O(*n*log*n*)) of constructive phase in Table 2. For the original implementation, "-" means that the running time exceeds 10 minutes and it cannot be recorded.

**Table 2:** Running time comparison.

Stochastic Problem	*n*	*W*	Running time (s)	
			Original implementation	Efficient implementation
P1-class1	10^1^	200	< 0.001	0.011
P2-class1	10^2^	500	0.009	0.017
P3-class1	10^3^	1000	0.722	0.073
P4-class1	10^4^	5000	67.984	0.681
P5-class1	10^5^	10000	-	8.218
P6-class1	10^6^	50000	-	103.382
P1-class2	10^1^	800	< 0.001	0.012
P2-class2	10^2^	2500	0.009	0.017
P3-class2	10^3^	8000	0.638	0.105
P4-class2	10^4^	25000	73.038	1.110
P5-class2	10^5^	80000	-	9.796
P6-class2	10^6^	250000	-	103.912
P1-class3	10^1^	2000	< 0.001	0.012
P2-class3	10^2^	5000	0.009	0.017
P3-class3	10^3^	10000	0.572	0.098
P4-class3	10^4^	50000	69.096	1.414
P5-class3	10^5^	100000	-	11.248
P6-class3	10^6^	500000	-	109.073

**Fig 7:**
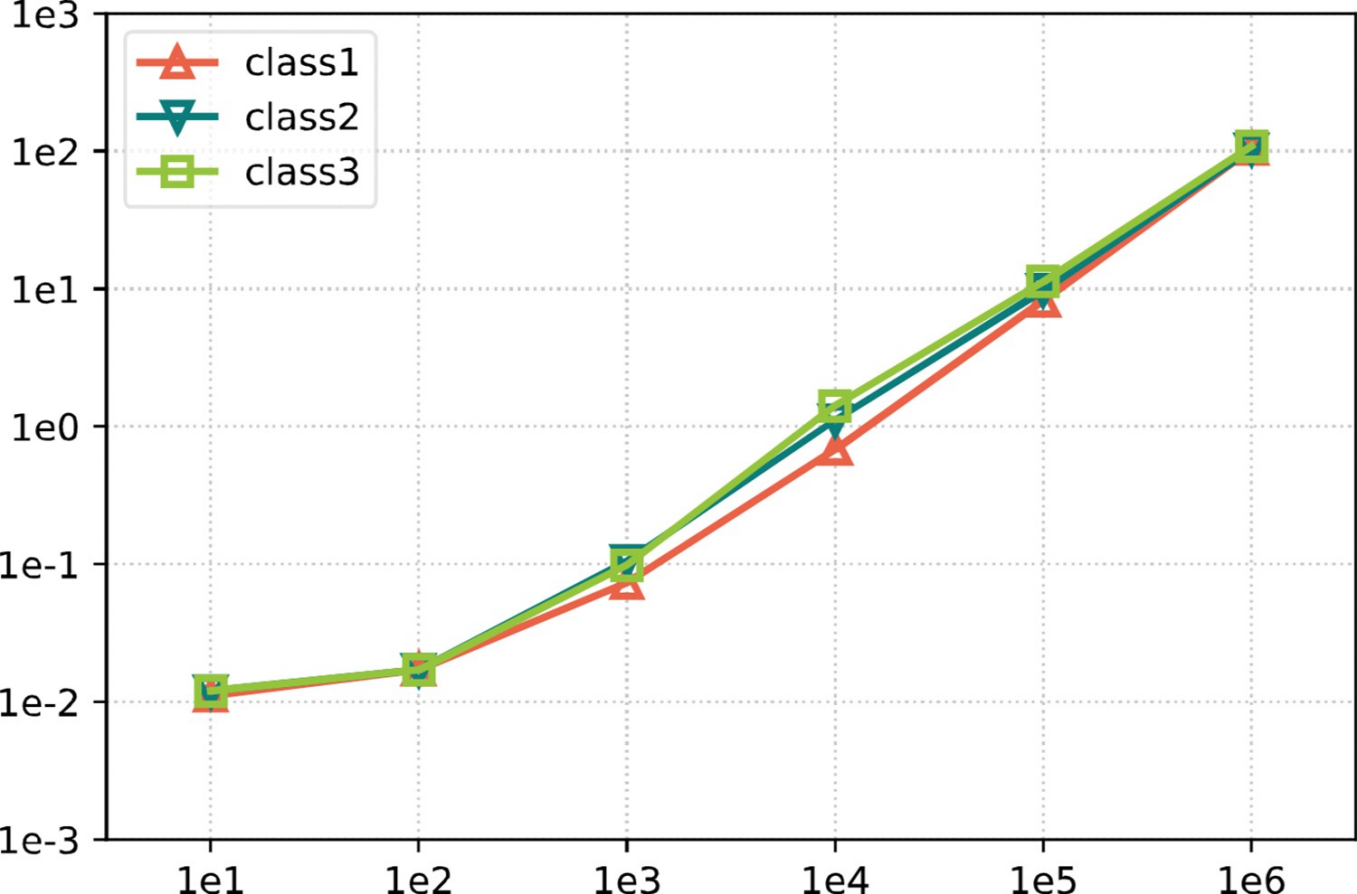
Efficient implementation of the constructive phase.

The time consumption for the efficient implementation is presented in Fig 7. The x-axis values denote the scale of the problem, and the y-axis values denote the running time (s),

The results from Table 2 and Fig 7 show that the proposed rectangular constructive algorithm is executed considerably fast. The efficient implementation takes almost the same amount of time compared with the original implementation on small-scale problems (n ≤ 10^2^), while it presents clear advantages over the original implementation on the problems with n ≥ 10^4^. The implementation of packing 10^4^ items would only takes about 1s, even for the extra-large-scale problem, the running time could be reduced to nearly 100s, which cannot be completed by the original implementation. We note that the ordinary behavior of the efficient implementation compared with the original implementation on small-scale problems could be explained by the preprocess procedure, since a certain amount of computational effort is allocated to the initialization of the designed data structures.

To test the performance concerning the packing qualities, we evaluate the proposed algorithm on the benchmark problem C21 [30], N13 [3], NT [31], Babu [32] and CX [33], same as the research in [34] has presented. For simplicity, we name the efficient constructive heuristic proposed in this paper (along with the packing optimization algorithm) as EH. The running time od EH is limited within 60s, and the optimization algorithm will be stopped when the packing length reaches *LB*. EH is run 10 times on each of these problems and the best result is recorded. The results are presented in Tables 3–6. The lower bound (optimal packing length) is denoted by *LB*, and the gap represents the result of 100 * (*L’*—*LB*) / *LB*.

**Table 3:** Results on problem C21.

			FH			BBFM			BFHA			EH		
	*n*	*LB*	*L’*	gap	time(s)	*L’*	gap	time(s)	*L’*	gap	time(s)	*L’*	gap	time(s)
C1P1	16	20	**20**	**0**	0	**20**	**0**	0	**20**	**0**	0	**20**	**0**	0.01
C1P2	17	20	**20**	**0**	0.01	21	5	0.29	**20**	**0**	0	**20**	**0**	0.01
C1P3	16	20	21	5	0	21	5	0.24	**20**	**0**	0	**20**	**0**	0.01
C2P1	25	15	16	6.7	0.02	16	6.7	0.54	**15**	**0**	0	**15**	**0**	0.08
C2P2	25	15	**15**	**0**	0	**15**	**0**	0.01	**15**	**0**	0	**15**	**0**	0.01
C2P3	25	15	**15**	**0**	0	16	6.7	0.59	**15**	**0**	0	**15**	**0**	0.01
C3P1	28	30	31	3.3	0.02	**30**	**0**	0.15	**30**	**0**	0	**30**	**0**	0.11
C3P2	29	30	31	3.3	0.02	31	3.3	0.73	**30**	**0**	0.21	**30**	**0**	1.18
C3P3	28	30	32	6.7	0.02	32	6.7	0.69	**30**	**0**	0.43	**30**	**0**	0.10
C4P1	49	60	61	1.7	0.16	62	3.3	2.33	**60**	**0**	25.18	**60**	**0**	0.85
C4P2	49	60	61	1.7	0.11	61	1.7	2.21	**60**	**0**	0.48	**60**	**0**	0.15
C4P3	49	60	61	1.7	0.08	61	1.7	2.21	**60**	**0**	1.71	**60**	**0**	0.32
C5P1	73	90	91	1.1	0.28	91	1.1	4.86	**90**	**0**	1.52	**90**	**0**	1.67
C5P2	73	90	**90**	**0**	0	91	1.1	4.84	**90**	**0**	0.89	**90**	**0**	1.29
C5P3	73	90	91	1.1	0.3	91	1.1	4.88	**90**	**0**	0.61	**90**	**0**	1.33
C6P1	97	120	121	0.8	0.83	121	0.8	8.63	**120**	**0**	113.17	**120**	**0**	9.64
C6P2	97	120	121	0.8	0.89	122	1.7	8.73	**120**	**0**	0.30	**120**	**0**	1.12
C6P3	97	120	121	0.8	0.76	121	0.8	8.76	**120**	**0**	21.78	**120**	**0**	10.78
C7P1	196	240	241	0.4	13.91	242	0.8	38.47	**240**	**0**	7411.88	241	0.4	60
C7P2	197	240	241	0.4	11.64	242	0.8	38.89	**240**	**0**	0.52	**240**	**0**	14.14
C7P3	196	240	241	0.4	15	241	0.8	38.79	**240**	**0**	2887.10	241	0.4	60

**Table 4:** Results on problem N13.

			FH			BBFM			BFHA			EH		
	*n*	*LB*	*L’*	gap	time(s)	*L’*	gap	time(s)	*L’*	gap	time(s)	*L’*	gap	time(s)
N1	10	40	**40**	**0**	0	**40**	**0**	0	**40**	**0**	0	**40**	**0**	0
N2	20	50	52	4	0	**50**	**0**	0.01	**50**	**0**	0.11	**50**	**0**	0.01
N3	30	50	51	2	0.02	52	4	0.76	**50**	**0**	0	**50**	**0**	0.07
N4	40	80	83	3.8	0.03	82	2.5	1.47	81	1.2	0.09	**80**	**0**	5.21
N5	50	100	102	2	0.08	102	2	2.36	101	1	10.92	**100**	**0**	1.90
N6	60	100	101	1	0.03	101	1	3.20	**100**	**0**	1.36	**100**	**0**	1.44
N7	70	100	102	2	0.13	105	5	4.60	**100**	**0**	50.37	**100**	**0**	16.96
N8	80	80	81	1.3	0.23	81	1.3	5.64	**80**	**0**	7.51	**80**	**0**	14.63
N9	100	150	151	0.7	0.14	151	0.7	9.18	**150**	**0**	21.70	**150**	**0**	5.32
N10	200	150	151	0.7	0.55	151	0.7	39.32	**150**	**0**	594.00	**150**	**0**	1.92
N11	300	150	151	0.7	1.48	**150**	**0**	15.30	**150**	**0**	1438.59	**150**	**0**	0.71
N12	500	300	301	0.3	5.63	302	0.7	282.63	301	0.3	5681.45	**300**	**0**	2.07
N13	3152	960	**960**	**0**	1.91	**960**	**0**	422.60	**960**	**0**	182.56	**960**	**0**	1.18

**Table 5:** Results on problem NT.

			BBF			IDBS			BFHA			EH		
	*n*	*LB*	*L’*	gap	time(s)	*L’*	gap	time(s)	*L’*	gap	time(s)	*L’*	gap	time(s)
NT-N-1	17	200	**-**	6	**-**	**-**	**0**	1.9	209.2	4.6	0.11	**200**	**0**	0.31
NT-N-2	25	200	**-**	6.5	**-**	**-**	1.8	37.14	209.8	4.9	0.19	205.8	2.9	60
NT-N-3	29	200	**-**	5	**-**	**-**	1.8	34.66	206.6	3.3	1.71	205.4	2.7	60
NT-N-4	49	200	**-**	2.5	**-**	**-**	1	35.24	205	2.5	2.24	203.8	1.9	60
NT-N-5	73	200	**-**	3.5	**-**	**-**	0.5	24.13	202.6	1.3	6.12	202.8	1.4	60
NT-N-6	97	200	**-**	2.5	**-**	**-**	0.5	0.47	201	0.5	15.63	201.8	0.9	60
NT-N-7	197	200	**-**	1.5	**-**	**-**	**0**	0.23	**200**	**0**	20.89	201	0.5	60
NT-T-1	17	200	**-**	**-**	**-**	**-**	0.5	1.37	211	5.5	0.1	201.6	0.8	25.38
NT-T-2	25	200	**-**	**-**	**-**	**-**	1.5	26.93	209.2	4.6	0.21	206	3	60
NT-T-3	29	200	**-**	**-**	**-**	**-**	1.7	48.83	207.2	3.6	1.37	206	3	60
NT-T-4	49	200	**-**	**-**	**-**	**-**	1.1	28.49	205.4	2.7	2.67	203.6	1.8	60
NT-T-5	73	200	**-**	**-**	**-**	**-**	0.5	20.55	202.6	1.3	8.32	202.8	1.4	60
NT-T-6	97	200	**-**	**-**	**-**	**-**	0.3	4.55	201	0.5	40.67	201.8	0.9	60
NT-T-7	199	200	**-**	**-**	**-**	**-**	**0**	5.63	200.4	0.2	136.52	201	0.5	60

**Table 6:** Results on problem Babu and CX.

			FH			IDBS			BFHA			EH		
	*n*	*LB*	*L’*	gap	time(s)	*L’*	gap	time(s)	*L’*	gap	time(s)	*L’*	gap	time(s)
Babu	50	375	-	-	-	**375**	**0**	0	**375**	**0**	0.10	**375**	**0**	0.01
50cx	50	600	624	4	0.33	601	0.2	11.43	604	0.7	41.38	612	2	60
100cx	100	600	619	3.2	3.56	607	1.2	59.31	610	1.7	228.24	606	1	60
500cx	500	600	**600**	**0**	2.00	**600**	**0**	2.62	**600**	**0**	410.33	601	0.2	60
1000cx	1000	600	**600**	**0**	0.02	**600**	**0**	0.86	**600**	**0**	16.92	**600**	**0**	1.85
5000cx	5000	600	**600**	**0**	0.05	**600**	**0**	2.23	**600**	**0**	837.11	**600**	**0**	0.32
10000cx	10000	600	**600**	**0**	0.05	**600**	**0**	5.98	**600**	**0**	2847.56	**600**	**0**	0.66
15000cx	15000	600	**600**	**0**	0.06	**600**	**0**	12.14	**600**	**0**	1986.6	**600**	**0**	1.03

Some excellent algorithms are listed here for comparison, they include FH (Leung et al., 2011, [35]), BBF (Aşık et al., 2009, [20]), BBFM (Özcan et al., 2013, [36]), IDBS (Wei et al., 2011, [19]), BFHA (Chen et al., 2019, [34]). To the best of our knowledge, IDBS performs rather effectively and it holds by far the best record on the rectangular strip packing problem with rotations. The optimal results (equal to *LB*) found by the above algorithms are presented in bold.

The computational results testify that EH behaves effectively on the benchmark problems, especially EH could find the optimal solutions on all the problems of N13 within 60s. On C21, EH appears slightly inferior to BFHA, but EH still provides 19 optimal results out of the 21 problems, while BFHA takes a large amount of computational effort on a few particular problems.

The problem NT has been relatively difficult to solve in the literature through heuristic approaches. IDBS shows rather excellent performance that it provides the minimum gap from the optimum, while EH behaves worse than IDBS and better than BFHA. We notice that EH could not provide a good result on the first problem of CX, this may be caused by the extreme shape of the rectangle and the placement strategy used in EH, i.e., the bottom left placement in this problem might generate some extreme gap, leading to the earlier placement of the extreme rectangle which would be later placed in the other position with a specific orientation. Then, vacant regions could be produced without filling, the layout would not be ideally compact.

Future research could examine the efficient implementation of more sophisticated placement strategy and evaluation rule for the item selection. Since the fitness evaluation adopted in this paper only presents the alignments between the edges, it could possibly be strengthened through an extended assessment on the discrepancy between the dimensions of the edges in the non-alignments.

## 5. Conclusions

We propose an efficient heuristic for the rectangular packing problem with rotations. The packing is comprised of the constructive phase and the improvement phase. We categorize the gaps on the skyline into three types according to their associated edges. Four data structures are designed to store the information used for item selection and a pointer is built to locate the minimum dimension item for the update of skyline. Through the designed data structures, the entire time complexity of the constructive phase is O(*n*log*n*). An optimization algorithm is presented for the improvement phase, where the packing is optimized through random swap on the sequence or the random change on the orientation of the item. We design three classes of stochastic problems with the number of items ranging from 10^1^ to 10^6^, the running time indicates the efficiency of the proposed algorithm. The proposed algorithm is also tested on the benchmark problem C21, N13, NT, Babu and CX, the results show that it provides satisfactory packing qualities.
